# *Mycobacterium decipiens* Infection in Patient Receiving Anti–TNF-α Therapy, France, 2024

**DOI:** 10.3201/eid3201.250518

**Published:** 2026-01

**Authors:** Justin Charles-Antoine Destoop, Corentin Poignon, William Danjou, Alexandre Bleibtreu, Valerie Pourcher, Gentiane Monsel

**Affiliations:** Pitié-Salpêtrière University Hospital, Paris, France (J.C.-A. Destoop, W. Danjou, A. Bleibtreu, V. Pourcher, G. Monsel); Dermatological Infectiology and Sexually Transmitted Infections Group of the French Society of Dermatology, Paris (J.C.-A. Destoop, G. Monsel); Sorbonne Université, Paris (C. Poignon); French National Institute of Health and Medical Research, Paris (C. Poignon, A. Bleibtreu, V. Pourcher)

**Keywords:** *Mycobacterium decipiens*, anti–TNF-α therapy, nontuberculous mycobacteria, tuberculosis and other mycobacteria, bacteria, tumor necrosis factor alpha, skin and soft tissue infections, travel, France

## Abstract

*Mycobacterium decipiens* is a newly identified species with high genomic similarity to *M. tuberculosis*. We report a cutaneous *M. decipiens* infection in a patient in France who had inflammatory bowel disease being treated with anti–tumor necrosis factor-α therapy. The infection was successfully treated with an oral antimicrobial regimen.

*Mycobacterium decipiens* is a recently identified nontuberculous mycobacterium genetically closest to the *M. tuberculosis* complex but of low virulence. Since its description in 2018, the medical literature documents 2 human infections (*1*,*2*). We report a cutaneous case of *M. decipiens* in a 60-year-old man living in an area between Paris and Corsica, France.

The patient sought treatment for skin lesions on his anterior right knee evolving over 9 months. His medical history included irritable bowel disease treated with the anti–tumor necrosis factor-α monoclonal antibody adalimumab (80 mg subcutaneously on the abdomen every 2 wks) for 9 years. Two months after a 2-week trip to French Guiana, where he hiked in forests and swam in rivers and pools, he noticed an erythematous papule that progressively worsened. He reported no history of trauma, sick contacts, or animal exposure and no adalimumab injections during the trip. He did report multiple mosquito bites. The man’s occupation involved building renovation and, on returning from French Guiana, he emptied stagnant swimming pool water in Corsica. 

Clinical examination revealed an erythematous, exudative, budding lesion over the patella, with multiple satellite nodules (Figure, panel A). Ipsilateral inguinal lymphadenopathy was nontender and subcentimetric. The patient reported no fever or weight loss. Magnetic resonance imaging of the right knee revealed superficial prepatellar bursitis and tendinopathy of the patellar and pes anserinus tendons. Blood counts and protein electrophoresis were unremarkable; C-reactive protein was moderately elevated at 25.6 mg/L (normal <5 mg/L). HIV serology result was negative. 

**Figure:**
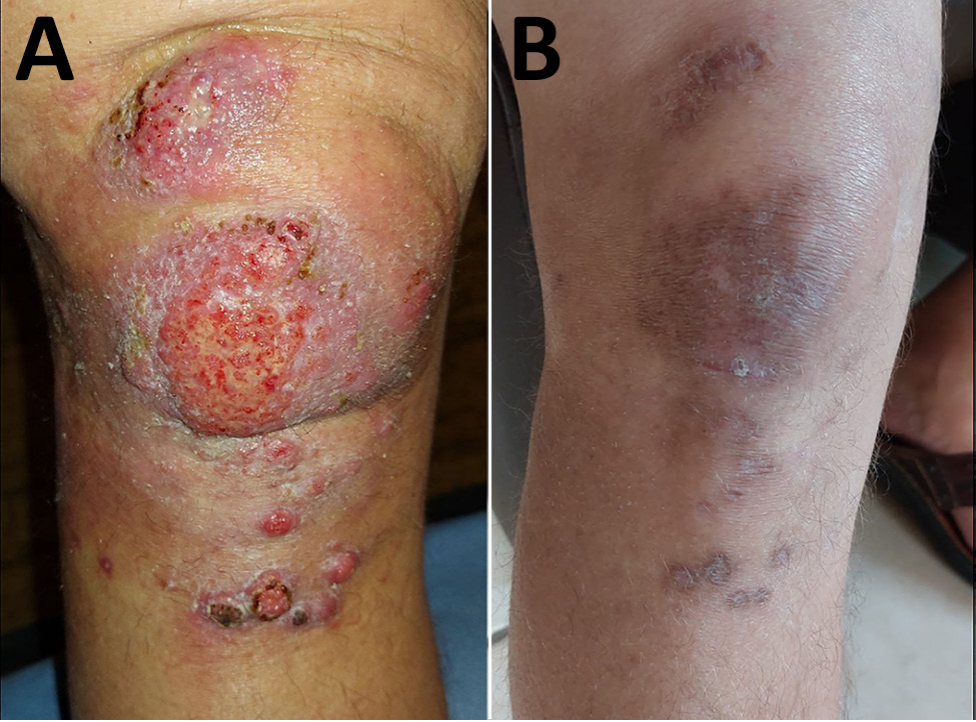
Cutaneous infection on the right knee before and after treatment from study of *Mycobacterium decipiens* infection in a patient treated with anti–tumor necrosis factor-α therapy, France, 2024. A) Before treatment: erythematous, exudative, budding lesion over the patella with multiple satellite nodules. B) After treatment: residual pigmented macular lesions, sometimes depressed, consistent with postinflammatory changes.

Histopathologic examination of a surgical biopsy showed epithelioid and giant cell granulomas without necrosis, along with superficial and deep lymphocytic inflammation around capillaries. Direct mycobacteriologic examination with auramine and Ziehl–Neelsen staining revealed 1–9 acid-fast bacilli per microscopic field. *M. tuberculosis* PCR (MDR/MTB ELITe MGB kit on ELITe InGenius platform; ELITechGroup; https://www.elitechgroup.com), targeting IS6110 and *rpoB*, produced negative results. Cultures produced dry, chamois-colored colonies on both liquid (mycobacteria growth indicator tube, modified 7H9) and solid (Coletsos) media. We observed growth after 24 days in the liquid medium and after 27 days on Coletsos medium at 30°C and 37°C; colonies were larger at 30°C. Commercial-line probe assays (GenoType CM and AS; Bruker; https://www.bruker.com) covering the most common and pathogenic nontuberculous mycobacterium as well as the *M. tuberculosis* complex failed to identify the isolate (*3*).

Sequencing of the 16S rRNA and *hsp65* genes showed 100% identity with *M. decipiens* (ATCC TSD-117; GenBank OY970459.1; TBL 1200985, Genbank NR_178632.1 for 16S and GenBank KJ371035.1 for hsp65). The closest non–M. decipiens matches were M. tuberculosis strains LP-0106963-RM2 (GenBank CP194255.1) and RM1 (GenBank CP194256.1), which showed 99.4% identity for 16S, and *M. szulgai* (GenBank KC481265.1, 96.2%) and *M. intracellulare* subspecies. *yongonense* (GenBank OR672012.1, 95.7%) for *hsp65*. Whole-genome sequencing confirmed the identification; analysis with KRAKEN2 using a dedicated mycobacterial reference database identified the isolate as *M. decipiens*, with 92.8% of reads assigned (*4*). We deposited raw reads in the National Center for Biotechnology Information under BioProject PRJNA1308041 (accession no. SRR35034371).

 We determined MICs for antimicrobial drugs potentially active against slow-growing mycobacteria using the SLOMYCO Sensititer system (Thermo Fisher Scientific; https://www.thermofisher.com) with Mueller–Hinton medium incubated at 30°C. MICs were readable after 10 days of incubation, and interpretation followed Clinical and Laboratory Standards Institute guidelines (*5*) for slowly growing mycobacteria other than *M. avium* and *M. kansasii*. The strain was susceptible to clarithromycin, rifabutin, moxifloxacin, amikacin, linezolid, and trimethoprim/sulfamethoxazole but resistant to rifampin.

The patient received empirical treatment with clarithromycin, ethambutol, and rifampin, resulting in poor response after 1 month. Once *M. decipiens* was identified, we replaced rifampin with moxifloxacin, leading to rapid clinical improvement within 4 weeks. We reduced adalimumab to 40 mg every 2 weeks. The patient continued on combination therapy for 2 additional months after complete remission. Reduction of adalimumab triggered an irritable bowel disease flare requiring corticosteroids and mesalazine enemas. Six months after discontinuation of antimicrobial drugs, only postinflammatory scarring remained (Figure, panel B).

*M. decipiens* is a slow-growing species, with optimal growth at 32°C–35°C. Colonies are rough, nonpigmented, and chamois-colored, resembling *M. tuberculosis*. Researchers have noted misidentification by rapid *M. tuberculosis* identification assays, complicating microbiologic diagnosis (*6*).

The reservoir of *M. decipiens* remains unknown. Reported cases of *M. decipiens* infection involved patients who had recently traveled to tropical regions (US Virgin Islands, Maldives, and French Guiana), suggesting a possible epidemiologic link (*1*,*2*). In this case, the patient reported river bathing in French Guiana and exposure to stagnant swimming pool water. In another case, the patient reported bathing in a tidal pool, raising the possibility of an aquatic reservoir (*1*). Reports have noted resistance of *M. decipiens* to first-line antituberculous drugs, including rifampin, isoniazid, and ethambutol (*6*). In our case, discontinuing rifampin and initiating moxifloxacin brought rapid clinical improvement. A prior reported case likewise involved administration of a macrolide-moxifloxacin combination with a third agent, which led to successful recovery (*1*).

In conclusion, clinical strains of *M. decipiens* in humans, as reported both here and in prior reports, appear to be susceptible to clarithromycin, moxifloxacin, linezolid, rifabutin, and trimethoprim/sulfamethoxazole (Table). Those agents should be considered in empirical regimens for possible *M. decipiens* infections, pending susceptibility results.

**Table:** Susceptibility of clinical isolates studied in prior reports and in our investigation of *Mycobacterium decipiens* infection in patient treated with anti–tumor necrosis factor-α therapy, France, 2024*

Antimicrobial drug	MIC of clinical isolates, μg/mL

Case 1 (*1*)	Case 2 (*2*)	Case 3 (this study)
Amikacin	≤1, S	4, S	1, S
Ciprofloxacin	4, R	1, S	4, R
Clarithromycin	4, S	0.25, S	8, S
Doxycycline	0.5, S	2, I	2, I
Ethambutol	4, I	8, R	4, I
Linezolid	≤1, S	≤1, S	1, S
Moxifloxacin	0.5, S	≤0.12, S	0.25, S
Rifabutin	≤0.25, S	≤0.25, S	<0.25, S
Rifampin	2, R	2, R	4, R
Trimethoprim/sulfamethoxazole	0.25/4.75, S	0.5/9.5, S	0.5/9.5, S

*The S/I/R breakpoints have been defined by analogy with the Clinical and Laboratory Standards Institute breakpoints for slowly growing mycobacteria other than *M. avium* and *M. kansasii* (*5*). I, intermediate; R, resistant; S, susceptible.
